# LUPUS PANNICULITIS AS AN INITIAL MANIFESTATION OF SYSTEMIC LUPUS ERYTHEMATOSUS

**DOI:** 10.4103/0019-5154.60364

**Published:** 2010

**Authors:** Raksha M Patel, Y S Marfatia

**Affiliations:** *From the Department of Skin V. D., Medical College, Vadodara - 390 001, Gujarat, India.*

**Keywords:** *Hydroxychloroquine*, *lupus panniculitis*, *SLE*

## Abstract

In May, 2003, a 28-year-old female presented with large non-healing ulcers on face, trunk and limbs covered with black hemorrhagic crust. There were no other systemic manifestations. Diagnosis of lupus panniculitis was considered on clinical and histopathological grounds. The lesions healed completely, with scarring, with systemic corticosteroid, hydroxychloroquine and topical 2% mupirocin. She came again in November, 2005, with malar rash, joint pain, scarring alopecia of the scalp and albuminuria. Her ANA, AntidsDNA came positive and diagnosed as having systemic lupus erythematosus (SLE). She responded well to systemic corticosteroid, antimalarial and topical antibacterial. The evolution of lupus panniculitis is slow and characterized by regression of the inflammatory lesions when treated with antimalarial drugs. The lupus panniculitis generally has a favorable course.

## Introduction

Lupus panniculitis is an unusual variant of lupus erythematosus. Most of the patients are adults in the age group of 20-60 years. Persistent, firm, well defined nodules and plaques on face, scalp, breast, arms, thighs, and buttocks characterize it generally, which may ulcerate and heal with scarring. Ulceration is rarely seen. Masood Q and Manzoor S reported two female cases of lupus erythematosus profundus in 1995.[1] Aggarwal *et al*. reported a case of lupus erythematosus profundus with associated mastitis but without any lesions of discoid lupus erythematosus or systemic lupus erythematosus.[2] Lupus panniculitis occurs in two to five per cent of SLE patients. Conversely, 10 to 15% of the patients with lupus panniculitis have or develop SLE. Lupus panniculitis was seen in six out of 228 DLE patients and four out of 86 in another series.[3]

## Case Report

A 28-year-old housewife presented with multiple large non-healing ulcers all over body since three months. She had undergone treatment from general practitioners and dermatologists without any significant clinical improvement.

She came to the Dermatology department in May, 2003, with history suggestive of asymptomatic nodules and plaque over the same site since four months. The lesions were initially small in size, rounded or oval in shape and firm in consistency; gradually increased in size to reach the present size and ulcerate. No history suggestive of purpuric lesion or associated systemic illness was found.

**Figure 1:**
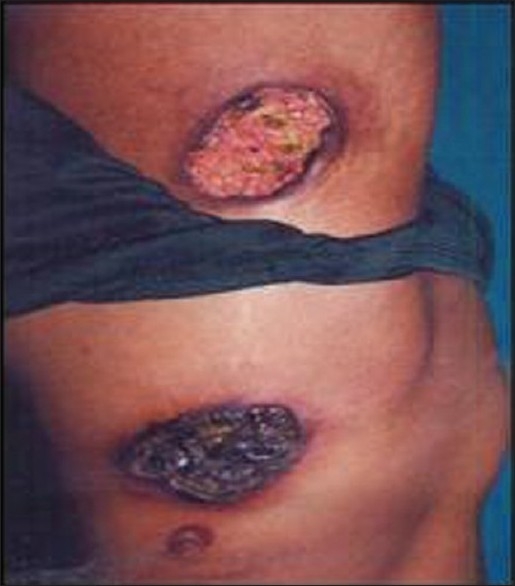
Lupus panniculitis before treatment

**Figure 2:**
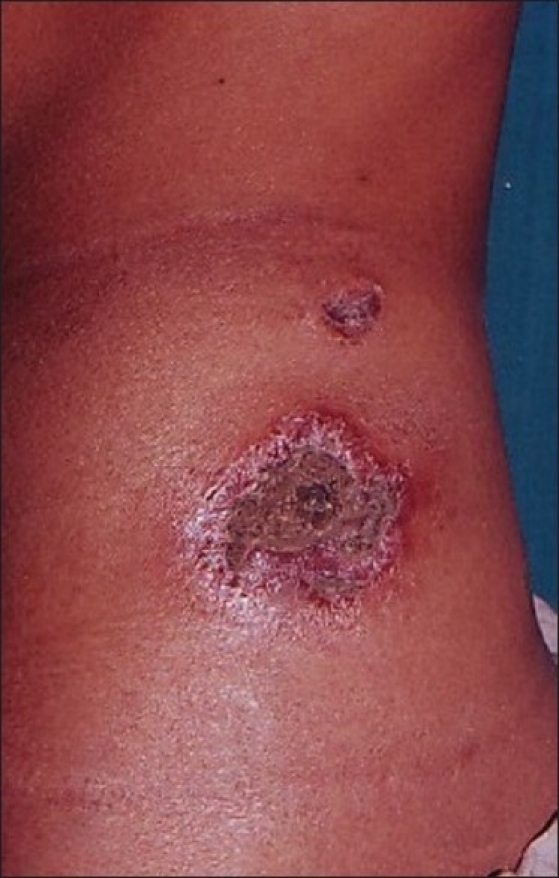
Lupus panniculitis before treatment

On examination, we found multiple, well defined, large deep ulcers, irregular in size and shape, with erythmatous raised edge, covered with yellow slough. Some lesions were covered with black hemorrhagic crust [Figures 1 and 2]. Diffuse loss of hair was present in our patient.

**Figure 3:**
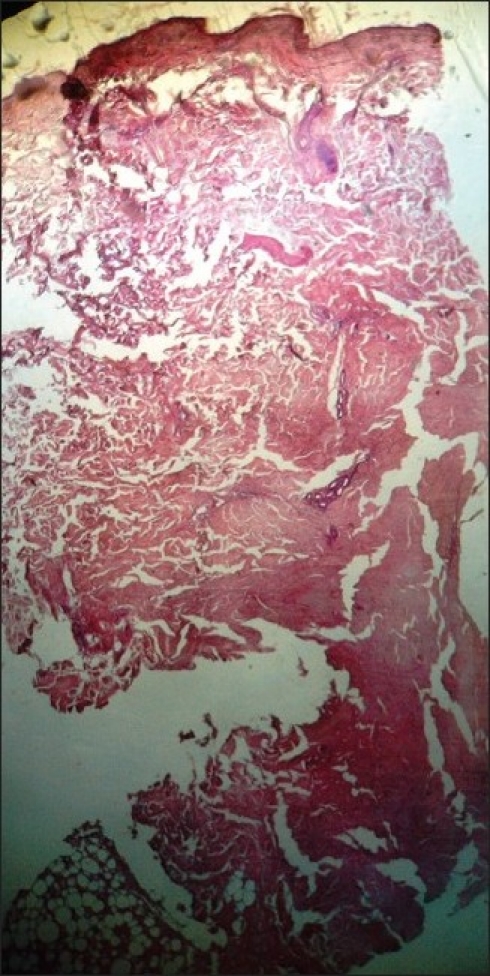
Vacuolar degeneration of basal cells and necrosis of fat lobules, inflammation of fibrous septa (H and E stain)

Provisional diagnosis of Pyoderma gangrenosum or Lupus panniculitis was considered. She was given systemic antibiotics, supportive treatment and local care of ulcer. Once infection was controlled, biopsy was taken from the active edge of the lesion and she was administered injection Dexamethasone 6 mg. intramuscularly, daily. Histopathologic section showed atrophic epidermis with focal basal vacuolation. Dermis showed edema with perivascular as well as intramural erythrocytic infiltration leading to concentric fibrosis of vessel wall. Subcutaneous tissue showed necrotic fat lobules containing hyalinized interstitial fibrin deposits and dense lymphocytic infiltration adjacent to necrotic zones, consistent with clinical diagnosis of Lupus panniculitis [Figure 3].

Besides routine investigations, special investigations, except anemia, like ANA, AntidsDNA, VDRL, and test for HIV antibodies were within normal limits. Direct immunoflorescence staining was not done because of non-affordability.

Hydroxychloroquine 200mg twice daily, orally, was added to systemic corticosteroid after ophthalmologic examination. Patient responded well to treatment. After 15 days, chloroquine phosphate 250mg, twice a day, was started in place of hydroxychloroquine to make it economical for the patient. All ulcers healed with scarring within two months and the patient was maintained on chloroquine phosphate 250 mg once daily and oral prednisolone in tapering dose. She came again in November, 2005, with malar rash, photosensitivity, joint pain, scarring alopecia and albuminuria. Her ANA, Anti-dsDNA were positive and then she was diagnosed with SLE.

She presented to us in March, 2006, with generalized edema and breathlessness. Skin lesions healed and photosensitivity improved. On USG abdomen and X-ray chest, she was found to have ascites, pleural effusion, and pericardial effusion. Patient was referred to the Medicine department for further management and advised to be admitted in ICCU, but she refused. Tuberculosis was ruled out by chest X-ray and examination of pleural fluid. Sugar, protein and ADA (Adenosine deaminase) levels in pleural fluid were within normal limits suggestive of non-infectious etiology.

She was started on prednisolone 40 mg, daily, in addition to chloroquine base 150 mg twice daily. She has not come again till date.

## Discussion

Lupus panniculitis is often difficult to diagnose as other form of panniculitis may present similarly. The knowledge of clinical features and histopathology of disease is important because lupus panniculitis may precede SLE by some years. Roughly 50% of patients with LE panniculitis have evidence of SLE.[4] Ajubi *et al*. described two patients in whom panniculitis was the first symptom of SLE[5] while in the Diaz-Jouanen *et al*. study three out of 270 patients of SLE were presented with panniculitis initially.[6] SLE should be considered an underlying cause in patients with panniculitis.

Pregnancy is not advised while using antimalarials and prefer to have them off the drugs for six months prior to conception due to long storage of drugs.[7] The antimalarials have several mechanisms of action. Fatigue, fever, headache, arthralgia, arthritis, pleuritis and pericardial inflammation may improve with the use of antimalarials.[7] It is generally agreed that no life threatening side effects are seen with presently used daily doses of antimalarials (chloroquine 250 mg or hydroxychloroquine 200-400 mg daily); there have been no controlled studies of antimalarials in SLE.[8] The incidence of site threatening retinopathy on hydroxychloroquine at the recommended dose of 400 mg daily is extremely small.[8] Hydroxychloroquine relieves the primary symptoms of SLE and also reduces the adverse effects of corticosteroid on lipoprotein metabolism. It has steroid sparing effects. Systemic corticosteroids should be reserved for widespread and resistant cases. Occasionally, in patients repeatedly losing hair, it is reasonable to give a short course of steroid at the very beginning of antimalarial therapy before they have had time to become efficacious.[7] Systemic corticosteroid are good alternative therapies for most of the cutaneous lupus erythematosus patients.[7] Methotrexate may be helpful adjuvant in patients unresponsive to antimalarials.[7] Success with Dapsone, Azathioprine, Cyclosporine, Cyclophosphamide has been described.[9] Surgical debridement and resection of individual lesions may be attended when all modalities fail.[9]

Interest in hydroxychloroquine has evolved from its role as a disease modifier to its role as a prophylactic agent against some of the major morbidities of SLE and its treatment, namely hyperlipidemia, diabetes mellitus and thrombosis. Given its low toxicity and high tolerability, its use as a “background medicine” in many SLE patients is considered as appropriate today as it was a decade ago.[10]
